# A Comparative Study on the Accuracy and Efficacy Between Dalton and CINtec^®^ PLUS p16/Ki-67 Dual Stain in Triaging HPV-Positive Women

**DOI:** 10.3389/fonc.2021.815213

**Published:** 2022-01-24

**Authors:** Ying Li, Yunfeng Fu, Bei Cheng, Xing Xie, Xinyu Wang

**Affiliations:** Department of Gynecologic Oncology, Women’s Hospital, School of Medicine, Zhejiang University, Hangzhou, China

**Keywords:** p16/Ki-67 dual stain, human papillomavirus, cervical cancer, cervical intraepithelial neoplasia, cervical cancer screening

## Abstract

**Background:**

CINtec^®^ PLUS p16/Ki-67 dual-stained cytology (DS) is an alternative test to cytology in triaging human papillomavirus (HPV)-positive women. Dalton p16/Ki-67 Dual Stain kit employs the similar immunocytochemical detection and operating procedures with CINtec^®^ PLUS, but its accuracy and efficacy in triaging HPV-positive women need to be evaluated.

**Methods:**

A total of 717 HPV-positive specimens of cervical exfoliated cells were included. Cytology, Dalton, and CINtec^®^ PLUS were subsequently performed, and two DS tests were separately completed in each of the same specimens. The results of two DS tests were head-to-head compared, and their efficacies to identify high-grade cervical intraepithelial neoplasia (CIN) were evaluated, using histopathology of biopsy as the golden standard.

**Results:**

The overall positive rate of two DS tests were 28.31% for Dalton and 33.89% for CINtec^®^ PLUS (p < 0.05); both rose with the increased severity of histopathological and cytological abnormalities. Compared to CINtec^®^ PLUS, the positive rate of Dalton was significantly lower in the normal histopathology group (p < 0.05) and lower, but not significantly, in mild abnormal histopathology and cytology NILM and LSIL groups. Two DS tests showed a good consistency (Kappa value, 0.63; 95% CI, 0.557–0.688), with 100% of consistency in the cytology HSIL group. Inconsistency occurred mainly in the cytology NILM and LSIL groups, with more Dalton negative but CINtec^®^ PLUS positive. Compared to CINtec^®^ PLUS, Dalton showed similar sensitivity (94.59% vs. 91.89%), but significantly higher specificity (75.29% vs. 69.26%, p = 0.013) and accuracy (76.29% vs. 70.43%, p = 0.012), with a larger area under the curve (AUC) of 0.849 (95% CI, 0.800–0.899) for identifying CIN3+. The similar results were observed when identifying CIN2+.

**Conclusions:**

Dalton presents the lower false positive rate and better efficacy in identifying high-grade CIN than CINtec^®^ PLUS, suggesting that Dalton may be superior to CINtec^®^ PLUS and an alternative technique for triaging primary HPV-positive women in cervical cancer screening.

## Introduction

Globally, cervical cancer remains the third most common malignancy in women, with approximately 601,000 new cases and 260,000 deaths annually (1). A large number of clinical trials and practices have shown that screening, using cytology and/or human papillomavirus (HPV) testing, is an effective way to reduce the incidence and mortality of cervical cancer. Within the last decade, the strategy of cervical cancer screening has been gradually shifting from primary cytology to primary HPV testing worldwide (2). Multiple professional societies, such as the American Cancer Society (ACS) (3), the American Society for Colposcopy and Cervical Pathology (ASCCP) (4), the European Society of Gynecologic Oncology and the European Federation of Colposcopy (ESGO-EFC) (5), and the US Preventive Services Task Force (USPSTF) (6), have recommended primary HPV testing to be preferred for cervical cancer screening. It has been proven that HPV testing is highly sensitive but lowly specific for identifying high-grade cervical intraepithelial neoplasia (CIN), especially in young women. Thus, HPV-positive women should be further triaged by another test to avoid unnecessary colposcopy referral.

Cytology is a preferred examination for triaging HPV-positive women because of its high specificity, but it is a subjective judgment, and the accuracy depends on the professional level of the cytologist (7). There is increasing evidence that p16/Ki-67 dual-stained cytology (DS) can be used as an alternative test for triaging HPV-positive women (8–10). Such a kind of DS tests can overcome the uncertainty of cytology through objective markers. In KPNC, ATHENA, and several other studies, DS test has been shown to have better performance compared to cytology for the detection of CIN3+/CIN2+ in HPV-positive women (11–16).

CINtec^®^ PLUS is one of the DS techniques specific to p16 and Ki-67, and its accuracy has been clinically and epidemiologically validated. CINtec^®^ PLUS has also been used as the comparator standard for evaluating various DS tests (17, 18). Dalton p16/Ki-67 Dual Stain kit is a product by Hangzhou Dalton Biosciences, China, which contains a mixture of mouse anti-human p16 antibody and rabbit anti-human Ki-67 antibody. The corresponding enzyme-labeled reagent of Dalton is a cocktail of horseradish peroxidase (HRP)-labeled goat anti-mouse IgG antibody and alkaline phosphatase (AP)-labeled goat anti-rabbit IgG antibody. Dalton also employs immunocytochemical detection and has similar operating procedures with CINtec^®^ PLUS, but its accuracy and efficacy for triaging HPV-positive women have not been evaluated up to date.

In this study, we separately performed Dalton and CINtec^®^ PLUS p16/Ki-67 dual stain in each of the same specimens of cervical exfoliated cells from 717 HPV-positive Chinese women, head-to-head compared the results of detection between two DS tests, and analyzed the efficacy of two DS tests in triaging HPV-positive women, using histopathology of biopsy as the golden standard. The aim of our study was to assess the value of Dalton p16/Ki-67 dual stain in triaging HPV-positive women.

## Subjects and Methods

### Subject Recruitment and Sample Collection

**Figure 1:**
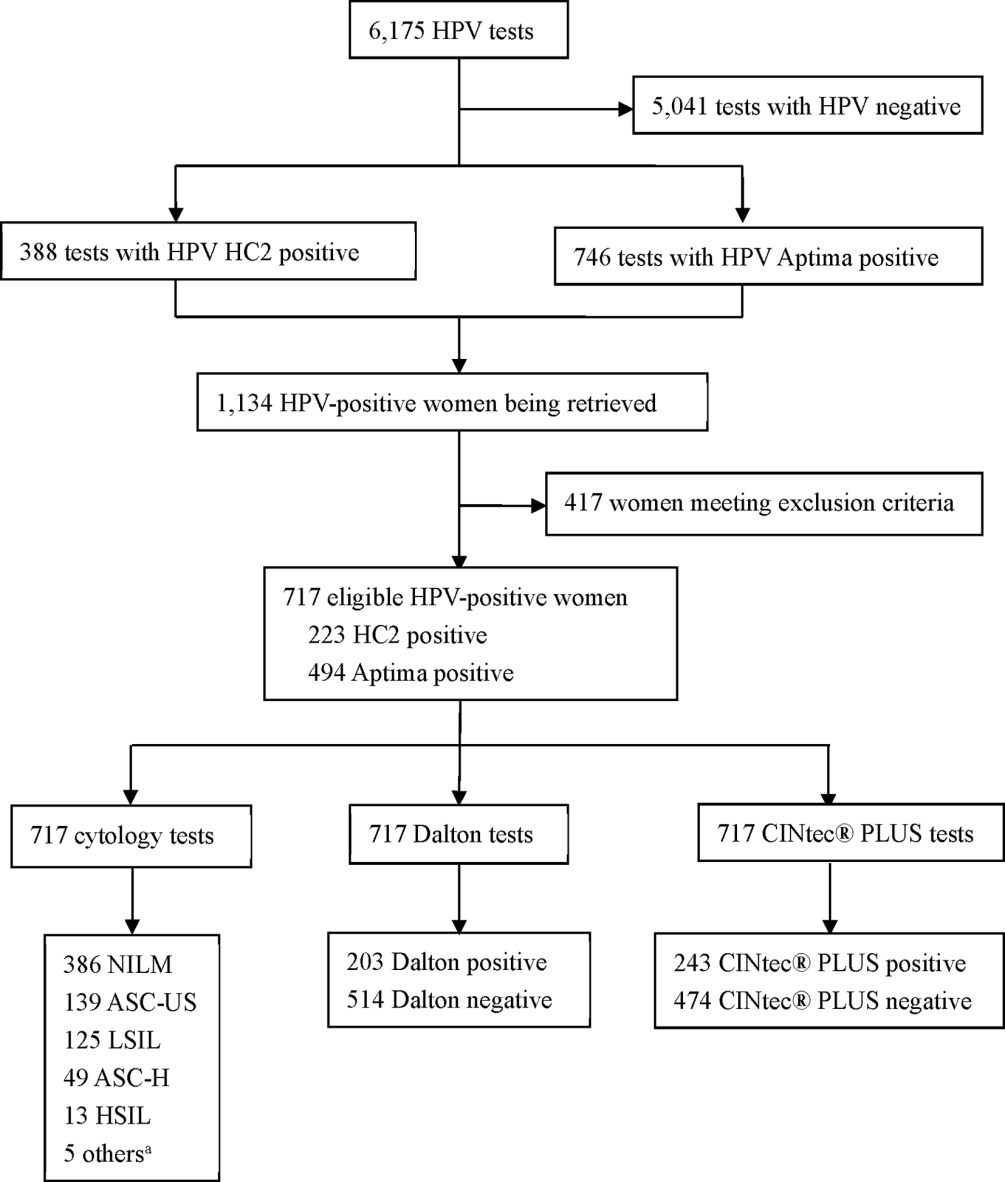
Flowchart of subject recruitment. HPV, human papillomavirus; NILM, negative for intraepithelial lesion or malignancy; ASC-US, atypical squamous cells of undetermined significance; LSIL, low-grade squamous intraepithelial lesion; ASC-H, atypical squamous cells, cannot rule out high-grade squamous intraepithelial lesion; HSIL, high-grade squamous intraepithelial lesions. ^a^Included 3 of SCC (squamous cell carcinoma), 1 of AC (adenocarcinoma) and 1 of AGC-NOS (atypical glandular cell of undetermined significance).

A total of 6,175 results of HPV testing were reviewed, which were from women who had received HPV testing in gynecological clinic of Women’s Hospital, School of Medicine, Zhejiang University, China, during September 2020 to May 2021, and those HPV-positive women were retrieved. The exclusion criteria from the study were ① previously confirmed CIN, cervical cancer, or other malignancies ②; previous therapeutic procedure to cervix ③; pregnancy ④; unsatisfactory sampling or insufficient amount of cells for test ⑤; no final histopathological diagnosis, and ⑥ refusing to sign informed consent. Finally, 717 HPV-positive women, aged 20–69 years (median age, 41 years), were included in this study (**Figure 1**). Written informed consents were obtained from all participants.

All subjects received either HPV Hybrid Capture 2 assay^®^ (HC2; Qiagen, Gaithersburg, MD) or Aptima^®^ HPV Test (Aptima; Hologic, San Diego, CA). HPV testing was performed following the manufacturer’s instructions. The recommended cutoff value is relative light units/cutoff (RLU/CO) ratio ≥1.0 for HC2 assay and signal/cutoff (S/CO) ratio ≥1.0 for Aptima assay. Residual specimens of cervical exfoliated cells after Aptima test were collected for further liquid-based cytology (LBC) (ThinPrep^®^; Hologic, Marlborough, MA) and two DS tests. While in women receiving HC2 test, cervical exfoliated cells were resampled for LBC and two DS tests. All LBC slides were evaluated by at least two pathologists; when inconsistent diagnosis occurred, the slide was read by the third pathologist, and the final diagnosis was made by two of them whose diagnoses were the same. The cytological findings were interpreted and categorized per the 2014 Bethesda System (19).

### p16/Ki-67 Dual Stain

**Figure 2:**
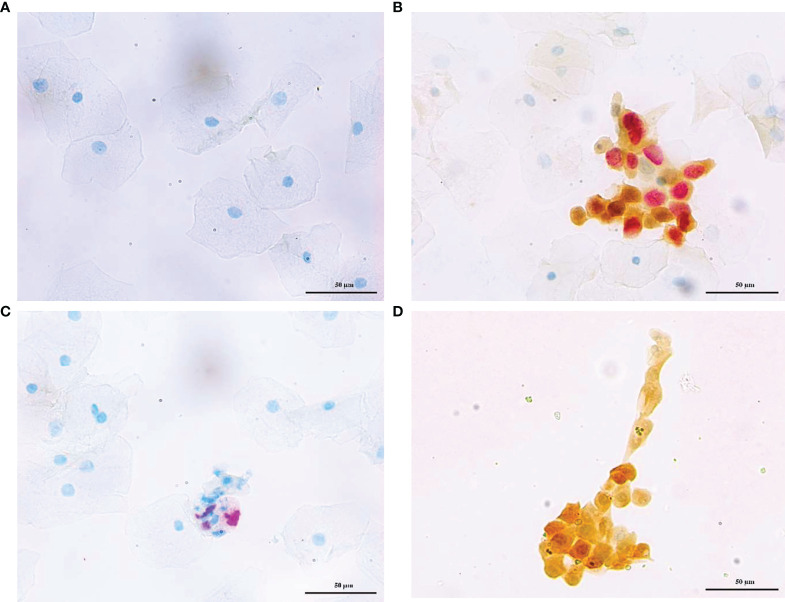
Representative photos of p16/Ki-67 dual stain cytology. Cervical exfoliated cells negative **(A)** and positive **(B)** for for Dalton, negative **(C)**, and positive **(D)** for CINtec^®^ PLUS, respectively. The brown cytoplasm/nuclear signal showed p16 staining alone, and the red nuclear signal showed Ki-67 staining alone. The positive p16/Ki-67 dual stain cell had both brown and red signal, which reflected the colocalization of p16 and Ki-67 in the same cell.

Dalton and CINtec^®^ PLUS p16/Ki-67 dual stain was separately completed in each of the same specimens of cervical exfoliated cells at a central laboratory (Dian Diagnostics Laboratories) using Dalton p16/Ki-67 Dual Stain kit (Dalton, Hangzhou, China) and CINtec^®^ PLUS Cytology kit (Roche mtm Laboratories AG, Mannheim, Germany), respectively. Dalton and CINtec^®^ PLUS DS slides were evaluated by two pathologists who were blind to each other. Positive p16/Ki-67 dual-stained cells were characterized by a brown cytoplasmic/nuclear signal for p16 overexpression and a red nuclear signal for Ki-67 expression within the same cell (**Figure 2**). The presence of at least one double-stained cell was sufficient to score the sample as positive, regardless of the morphological appearance (20). All specimens were conducted per the manufacturer’s instructions and stored at room temperature.

### Colposcopy and Histopathological Examination

All HPV-positive women in this study underwent colposcopy with at least one biopsy taken according to colposcopy images, and most of them received multiple biopsies in order to improve the detection rate for CIN (21, 22). Final diagnoses were made by histopathological findings classified according to the CIN nomenclature (23).

### Statistical Analysis

The chi-square test was used to compare proportions among different groups. Cohen’s kappa was calculated to estimate interobserver consistency between Dalton and CINtec^®^ PLUS. Categorical variables [sensitivity, specificity, positive predictive value (PPV), negative predictive value (NPV), and accuracy] were summarized using percentages and 95% confidence interval (CI). Likelihood ratio positive (LR+), likelihood ratio negative (LR−), and the receiver operating characteristic (ROC) curve with 95% CI were used to assess the efficacy in detecting high-grade lesions (including CIN3+ and CIN2+). Data were analyzed with software SPSS21.0 and VassarStats (online). All statistical tests were two-sided, and the value of p < 0.05 was considered statistically significant.

## Results

Of the 717 HPV-positive women, 223 (31.10%) received HC2 assay, and other 494 (68.90%) received Aptima assay. For cytology, 331 (46.16%) were abnormal, including 139 (41.99%) of ASC-US (atypical squamous cells of undetermined significance), 125 (37.77%) of LSIL (low-grade squamous intraepithelial lesion), 49 (14.80%) of ASC-H (atypical squamous cells, cannot rule out high-grade squamous intraepithelial lesion), 13 (3.93%) of HSIL (high-grade squamous intraepithelial lesions), 3 (0.91%) of SCC (squamous cell carcinoma), 1 (0.30%) of AC (adenocarcinoma), and 1 (0.30%) of AGC-NOS (atypical glandular cell of undetermined significance). The remaining 386 specimens were NILM (negative for intraepithelial lesion or malignancy) (**Figure 1**). The final histopathological diagnoses of all subjects were 468 (65.27%) of normal, 169 (23.57%) of CIN1, 43 (6.00%) of CIN2, 29 (4.04%) of CIN3, and 8 (1.12%) of invasive cancer, including 6 squamous cell carcinomas and 2 adenocarcinomas.

Dual-stained cytology for p16/Ki-67 was separately performed by Dalton and CINtec^®^ PLUS test in the same sample, and the representative stains of two DS tests were displayed in **Figure 2**. The overall positive rates of DS were 28.31% (203/717) for Dalton and 33.89% (243/717) for CINtec^®^ PLUS, with a significant difference between them (p < 0.05) (**Table 1**). **Tables 1** and **2** displayed the positive distribution of DS by Dalton and CINtec^®^ PLUS in different histopathological and cytological subgroups, respectively. The DS positive rates of both tests rose with the increased severity of histopathological and cytological abnormalities. Compared to CINtec^®^ PLUS, the positive rate of Dalton was significantly lower in the normal histopathology group (p < 0.05) and slightly lower in mild abnormal histopathology and cytology NILM and LSIL groups.

**Table 1:** The positivity of Dalton and CINtec^®^ PLUS p16/Ki-67 Dual Stain in different histopathology groups.

Histology	Total	Dalton+	CINtec^®^ PLUS+	*p*-value
	N	N (%)	N (%)	
Normal	468	84 (17.95%)	109 (23.29%)	0.043^a^
CIN1	169	49 (28.99%)	64 (37.87%)	0.084
CIN2	43	35 (81.40%)	36 (83.72%)	0.776
CIN3	29	27 (93.10%)	27 (93.10%)	1.000
Cancer	8	8 (100.00%)	7 (87.50%)	<0.001^a^
Total	717	203 (28.31%)	243 (33.89%)	0.022^a^

CIN, cervical intraepithelial neoplasia.

a A significance between groups (p < 0.05).

**Table 2:** The positivity of Dalton and CINtec^®^ PLUS p16/Ki-67 Dual Stain in different cytology groups.

Cytology	Total	Dalton+	CINtec^®^ PLUS+	*p*-value
	N	N (%)	N (%)	
NILM	386	76 (19.69%)	96 (24.87%)	0.084
ASC-US	139	41 (29.50%)	43 (30.94%)	0.794
LSIL	125	36 (28.80%)	50 (40.00%)	0.062
ASC-H	49	33 (67.35%)	37 (75.51%)	0.371
HSIL	13	12 (92.31%)	12 (92.31%)	1.000
Others^a^	5	5 (100%)	5 (100%)	1.000

NILM, negative for intraepithelial lesion or malignancy; ASC-US, atypical squamous cells of undetermined significance; LSIL, low-grade squamous intraepithelial lesion; ASC-H, atypical squamous cells, cannot rule out high-grade squamous intraepithelial lesion; HSIL, high-grade squamous intraepithelial lesions.

a Included 3 of SCC (squamous cell carcinoma), 1 of AC (adenocarcinoma) and 1 of AGC-NOS (atypical glandular cell of undetermined significance).

Totally, 37 specimens were Dalton positive but CINtec^®^ PLUS negative, while 77 were Dalton negative but CINtec^®^ PLUS positive, as shown in **Table 3**. A good consistency was found between Dalton and CINtec^®^ PLUS (Kappa value, 0.63; 95% CI, 0.557–0.688). Further analysis demonstrated complete consistency between two tests in the cytology HSIL group, and inconsistency mainly occurred in the cytology NILM and LSIL groups, with more Dalton negative but CINtec^®^ PLUS positive, implying that Dalton may possess the potential of lower false positive rate than CINtec^®^ PLUS in identifying high-grade lesions.

**Table 3:** Disagreement between Dalton and CINtec^®^ PLUS p16/Ki-67 Dual Stain in different cytology groups^a,b^.

	NILM	ASC-US	LSIL	ASC-H	HSIL	Other	Total
Dalton(+)/CINtec^®^ PLUS(−)	17	6	12	2	0	0	37
Dalton(−)/CINtec^®^ PLUS(+)	37	8	26	6	0	0	77

a Data were presented as case number.

b kappa value = 0.63, 95% confidence intervals (CI) = 0.557–0.688.

NILM, negative for intraepithelial lesion or malignancy; ASC-US, atypical squamous cells of undetermined significance; LSIL, low-grade squamous intraepithelial lesion; ASC-H, atypical squamous cells, cannot rule out high-grade squamous intraepithelial lesion; HSIL, high-grade squamous intraepithelial lesions.

**Figure 3:**
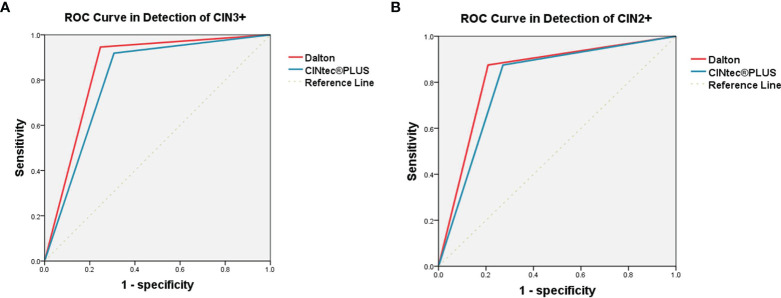
ROC curve analysis of Dalton and CINtec^®^ PLUS in detecting CIN3+ **(A)** and CIN2+ **(B)**. **(A)** The AUC were 0.849 (95% CI, 0.800-0.899) for Dalton and 0.806 (95% CI, 0.747-0.865) for CINtec^®^ PLUS. **(B)** The AUC were 0.833 (95% CI, 0.787-0.879) for Dalton and 0.802 (95% CI, 0.754-0.849) for CINtec^®^ PLUS. ROC, receiver operating characteristic; CIN3+, cervical intraepithelial neoplasia grade 3 or worse; CIN2+, cervical intraepithelial neoplasia grade 2 or worse; AUC, area under the curve.

The efficacy was further compared between Dalton and CINtec^®^ PLUS for predicting high-grade CIN in HPV-positive women (**Table 4** and **Figure 3**). In identifying CIN3+, the sensitivity, specificity, PPV, NPV, and accuracy of Dalton were 94.59%, 75.29%, 17.24%, 99.61%, and 76.29%, respectively. Among those, the specificity and accuracy of Dalton were significantly higher than those of CINtec^®^ PLUS (both p < 0.05). Similar results were observed when identifying CIN2+. ROC curves showed that Dalton occupied a larger area under the curve (AUC) in predicting both CIN3+ (0.849; 95% CI, 0.800–0.899) and CIN2+ (0.833; 95% CI, 0.787–0.879). Our results together suggest that Dalton presents the better efficacy than CINtec^®^ PLUS for identifying high-grade CIN in triaging HPV-positive women.

**Table 4:** Efficacy of Dalton and CINtec^®^ PLUS p16/Ki-67 Dual Stain to identify CIN3+ and CIN2+ among 717 HPV-positive women.

Test	Dalton^a^	CINtec^®^ PLUS^a^	*p*-value
Detection of CIN3+ (n = 37)			
Sensitivity	94.59% (82.47% to 99.06%)	91.89% (76.98% to 97.88%)	1.000
Specificity	75.29% (71.84% to 78.46%)	69.26% (65.62% to 72.69%)	0.013^b^
PPV	17.24% (12.45% to 23.30%)	13.99% (10.01% to 19.14%)	0.345
NPV	99.61% (98.44% to 99.93%)	99.37% (98.00% to 99.83%)	0.675
Accuracy	76.29% (73.04% to 79.26%)	70.43% (66.99% to 73.65%)	0.012^b^
LR+	3.83 (3.29 to 4.46)	2.99 (2.58 to 3.47)	
LR-	0.07 (0.02 to 0.28)	0.12 (0.04 to 0.35)	
Detection of CIN2+ (n = 80)			
Sensitivity	87.50% (77.76% to 93.52%)	87.50% (77.76% to 93.52%)	1.000
Specificity	79.12% (75.71% to 82.17%)	72.84% (69.18% to 76.23%)	0.009^b^
PPV	34.48% (28.06% to 41.50%)	28.81% (23.29% to 35.01%)	0.198
NPV	98.05% (96.33% to 99.00%)	97.89% (96.03% to 98.92%)	0.855
Accuracy	80.06% (76.98% to 82.82%)	74.48% (71.16% to 77.53%)	0.012^b^
LR+	4.19 (3.53 to 4.98)	3.22 (2.77 to 3.75)	
LR-	0.16 (0.09 to 0.28)	0.17 (0.10 to 0.31)	

a Data were presented % or value, 95% confidence intervals (CI).

b A significance between groups (p < 0.05).

CIN3+, cervical intraepithelial neoplasia grade 3 or worse; CIN2+, cervical intraepithelial neoplasia grade 2 or worse; HPV, human papillomavirus; PPV, positive predictive value; NPV, negative predictive value; LR+, likelihood ratio-positive; LR-, likelihood ratio–negative.

## Discussion

Dual stain for p16/Ki-67 is a kind of technique using immunohistochemistry specific to p16 and Ki-67, respectively. The p16, a cyclin-dependent kinase inhibitor, acts as a tumor suppressor in most cells (24), but HPV E7 oncoprotein mediates the degradation of retinoblastoma protein (Rb), and p16 exhibits oncogenic activity in HPV-transformed cervical cancer cells (25). Ki-67 is a positive marker for cell proliferation (26). In normal conditions, they usually do not co-express in the same cervical epithelial cell. Co-expression of two molecules indicates the deregulation of the cell cycle mediated by infected high-risk HPV and suggests the possibility of high-grade CIN (8).

Previous studies revealed that CINtec^®^ PLUS possessed improved performance, compared to cytology, for triaging HPV-positive women (11, 13, 15). CINtec^®^ PLUS Cytology Test is an assay approved by the Food and Drug Administration (FDA, USA) (https://www.fda.gov/media/136682/download) and has been regarded as the comparator standard for evaluating various tests (17, 18, 27). However, no comparative study between CINtec^®^ PLUS and other p16/Ki-67 DS stain test has been reported up to date. In this study, we head-to-head compared the DS results between Dalton and CINtec^®^ PLUS in the same specimens from 717 HPV-positive women and found a good consistency between two tests with a kappa value of 0.63. Especially in the cytology HSIL group, two tests showed complete consistency. Inconsistency occurred mainly in the cytology NILM and LSIL groups, which showed more Dalton negative but CINtec^®^ PLUS positive, combined with the fact that the positive rate of Dalton was much lower than that of CINtec^®^ PLUS in normal and mild abnormal cytology groups, suggesting that Dalton may have the lower probability of false positive rate than CINtec^®^ PLUS. A possible explanation for this phenomenon was that Dalton Ki-67 antibody displayed better visibility in DS-positive cells (**Figure 2B**), which might reduce the ambiguous judgment of the pathologist.

Because of the low specificity of HPV test, a highly specific triage test is very important to reduce colposcopy referral in primary HPV screening. In view of its high specificity, cytology has been recommended as the preferred method for triaging HPV-positive women (5). Previous studies demonstrated that CINtec^®^ PLUS had higher sensitivity, but similar specificity, than cytology (11, 13, 15). Thus, it seems that the specificity of CINtec^®^ PLUS needs to be improved. In this study, we compared the efficacy between Dalton and CINtec^®^ PLUS in predicting high-grade CIN and found that Dalton showed the significantly higher specificity (75.29% vs. 69.26%, p = 0.013) and accuracy (76.29% vs. 70.43%, p = 0.012) than CINtec^®^ PLUS, with the equal sensitivity for identifying CIN3+, and the results were similar for identifying CIN2+. Moreover, we observed that Dalton occupied a larger AUC than CINtec^®^ PLUS in identifying both CIN3+ and CIN2+. Our results together suggested that Dalton indeed increased the specificity but did not decrease the sensitivity, compared to CINtec^®^ PLUS, in identifying high-grade CIN. Hence, Dalton may be a superior test to CINtec^®^ PLUS for triaging HPV-positive women.

There were still some limitations of our study. All the participants came from a gynecological clinic; the proportions of CIN or above and high-grade CIN or above were 34.73% (249/717) and 11.16% (80/717), respectively, in our series, both appeared to be higher than those in the general population, which implies that there may be bias in our samples. In addition, our cross-sectional study cannot evaluate the long-term risk of high-grade lesions in those Dalton-negative women. Therefore, a longitudinal study on Dalton is needed.

In summary, we evaluated Dalton and CINtec^®^ PLUS as triage tests in 717 HPV-positive women and observed a good consistency between the two tests. Inconsistency mainly occurred in normal and mild abnormal cytology with more Dalton negative but CINtec^®^ PLUS positive. Using histopathological diagnosis of the biopsy as the golden standard, Dalton showed better efficacy than CINtec^®^ PLUS with higher specificity and similar sensitivity in identifying high-grade CIN. Our results suggest that Dalton may be superior to CINtec^®^ PLUS in identifying high-grade CIN and is an alternative technique for triaging primary HPV-positive women in cervical cancer screening.

## Data Availability Statement

The original contributions presented in the study are included in the article/supplementary material. Further inquiries can be directed to the corresponding author.

## Ethics Statement

The studies involving human participants were reviewed and approved by the Ethics Committees of Women’s Hospital, School of Medicine, Zhejiang University. The patients/participants provided their written informed consent to participate in this study.

## Author Contributions

YL, YF, and BC had full access to all the data in the study and took responsibility for the integrity of the data and accuracy of the data analysis. XX and XW designed and organized the study. All authors contributed to the article and approved the submitted version.

## Funding

This study was supported by the Key Research and Development Project of Zhejiang Province (2020C03025).

## Conflict of Interest

The authors declare that the research was conducted in the absence of any commercial or financial relationships that could be construed as a potential conflict of interest.

## Publisher’s Note

All claims expressed in this article are solely those of the authors and do not necessarily represent those of their affiliated organizations, or those of the publisher, the editors and the reviewers. Any product that may be evaluated in this article, or claim that may be made by its manufacturer, is not guaranteed or endorsed by the publisher.

## References

[1] Global Burden of Disease Cancer C Fitzmaurice C Abate D Abbasi N Abbastabar H Abd-Allah F. Global, Regional, and National Cancer Incidence, Mortality, Years of Life Lost, Years Lived With Disability, and Disability-Adjusted Life-Years for 29 Cancer Groups, 1990 to 2017: A Systematic Analysis for the Global Burden of Disease Study. JAMA Oncol (2019) 5(12):1749–68. doi: 10.1001/jamaoncol.2019.2996 PMC6777271 31560378

[2] Wentzensen N Arbyn M Berkhof J Bower M Canfell K Einstein M. Eurogin 2016 Roadmap: How HPV Knowledge Is Changing Screening Practice. Int J Cancer (2017) 140(10):2192–200. doi: 10.1002/ijc.30579 28006858

[3] Fontham ETH Wolf AMD Church TR Etzioni R Flowers CR Herzig A. Cervical Cancer Screening for Individuals at Average Risk: 2020 Guideline Update From the American Cancer Society. CA Cancer J Clin (2020) 70(5):321–46. doi: 10.3322/caac.21628 32729638

[4] Huh WK Ault KA Chelmow D Davey DD Goulart RA Garcia FAR. Use of Primary High-Risk Human Papillomavirus Testing for Cervical Cancer Screening: Interim Clinical Guidance. Obstet Gynecol (2015) 125(2):330–37. doi: 10.1097/AOG.0000000000000669 25569009

[5] Kyrgiou M Arbyn M Bergeron C Bosch FX Dillner J Jit M. Cervical Screening: ESGO-EFC Position Paper of the European Society of Gynaecologic Oncology (ESGO) and the European Federation of Colposcopy (EFC). Br J Cancer (2020) 123(4):510–17. doi: 10.1038/s41416-020-0920-9 PMC7434873 32507855

[6] Force USPST Curry SJ Krist AH Owens DK Barry MJ Caughey AB. Screening for Cervical Cancer: US Preventive Services Task Force Recommendation Statement. JAMA (2018) 320(7):674–86. doi: 10.1001/jama.2018.10897 30140884

[7] Ebisch RM Siebers AG Bosgraaf RP Massuger LF Bekkers RL Melchers WJ. Triage of High-Risk HPV Positive Women in Cervical Cancer Screening. Expert Rev Anticancer Ther (2016) 16(10):1073–85. doi: 10.1080/14737140.2016.1232166 27598683

[8] Yu L Fei L Liu X Pi X Wang L Chen S. Application of P16/Ki-67 Dual-Staining Cytology in Cervical Cancers. J Cancer (2019) 10(12):2654–60. doi: 10.7150/jca.32743 PMC6584925 31258773

[9] Wright TC Jr Stoler MH Ranger-Moore J Fang Q Volkir P Safaeian M. Clinical Validation of P16/Ki-67 Dual-Stained Cytology Triage of HPV-Positive Women: Results From the IMPACT Trial. Int J Cancer (2022) 150(3):461–71. doi: 10.1002/ijc.33812 PMC9293341 34536311

[10] Li YC Zhao YQ Li TY Chen W Liao GD Wang HR. The Performance of Immunocytochemistry Staining as Triaging Tests for High-Risk HPV-Positive Women: A 24-Month Prospective Study. J Oncol (2020) 2020:6878761. doi: 10.1155/2020/6878761 32565806 PMC7271243

[11] Gustinucci D Giorgi Rossi P Cesarini E Broccolini M Bulletti S Carlani A. Use of Cytology, E6/E7 mRNA, and P16ink4a-Ki-67 to Define the Management of Human Papillomavirus (HPV)-Positive Women in Cervical Cancer Screening. Am J Clin Pathol (2016) 145(1):35–45. doi: 10.1093/ajcp/aqv019 26712869

[12] Clarke MA Cheung LC Castle PE Schiffman M Tokugawa D Poitras N. Five-Year Risk of Cervical Precancer Following P16/Ki-67 Dual-Stain Triage of HPV-Positive Women. JAMA Oncol (2019) 5(2):181–86. doi: 10.1001/jamaoncol.2018.4270 PMC6439556 30325982

[13] Wright TC Jr Behrens CM Ranger-Moore J Rehm S Sharma A Stoler MH. Triaging HPV-Positive Women With P16/Ki-67 Dual-Stained Cytology: Results From a Sub-Study Nested Into the ATHENA Trial. Gynecol Oncol (2017) 144(1):51–6. doi: 10.1016/j.ygyno.2016.10.031 28094038

[14] Stoler MH Baker E Boyle S Aslam S Ridder R Huh WK. Approaches to Triage Optimization in HPV Primary Screening: Extended Genotyping and P16/Ki-67 Dual-Stained Cytology-Retrospective Insights From ATHENA. Int J Cancer (2020) 146(9):2599–607. doi: 10.1002/ijc.32669 PMC7078939 31490545

[15] Ikenberg H Bergeron C Schmidt D Griesser H Alameda F Angeloni C. Screening for Cervical Cancer Precursors With P16/Ki-67 Dual-Stained Cytology: Results of the PALMS Study. J Natl Cancer Inst (2013) 105(20):1550–7. doi: 10.1093/jnci/djt235 PMC3814411 24096620

[16] Wentzensen N Fetterman B Castle PE Schiffman M Wood SN Stiemerling E. P16/Ki-67 Dual Stain Cytology for Detection of Cervical Precancer in HPV-Positive Women. J Natl Cancer Inst (2015) 107(12):djv257. doi: 10.1093/jnci/djv257 26376685 PMC4675094

[17] Szekerczes T Galamb A Kocsis A Benczik M Takacs T Martonos A. Dual-Stained Cervical Cytology and Histology With Claudin-1 and Ki67. Pathol Oncol Res (2019) 25(2):477–86. doi: 10.1007/s12253-018-0384-x 29442221

[18] Benczik M Galamb A Koiss R Kovacs A Jaray B Szekely T. Claudin-1 as a Biomarker of Cervical Cytology and Histology. Pathol Oncol Res (2016) 22(1):179–88. doi: 10.1007/s12253-015-9990-z 26463354

[19] Nayar R Wilbur DC. The Pap Test and Bethesda 2014. Cancer Cytopathol (2015) 123(5):271–81. doi: 10.1002/cncy.21521 25931431

[20] Wentzensen N Schwartz L Zuna RE Smith K Mathews C Gold MA. Performance of P16/Ki-67 Immunostaining to Detect Cervical Cancer Precursors in a Colposcopy Referral Population. Clin Cancer Res (2012) 18(15):4154–62. doi: 10.1158/1078-0432.CCR-12-0270 PMC4237612 22675168

[21] Wentzensen N Walker JL Gold MA Smith KM Zuna RE Mathews C. Multiple Biopsies and Detection of Cervical Cancer Precursors at Colposcopy. J Clin Oncol (2015) 33(1):83–9. doi: 10.1200/JCO.2014.55.9948 PMC4268255 25422481

[22] Perkins RB Guido RS Castle PE Chelmow D Einstein MH Garcia F. ASCCP Risk-Based Management Consensus Guidelines for Abnormal Cervical Cancer Screening Tests and Cancer Precursors. J Low Genit Tract Dis (2020) 24(2):102–31. doi: 10.1097/LGT.0000000000000525 PMC7147428 32243307

[23] Darragh TM Colgan TJ Cox JT Heller DS Henry MR Luff RD. The Lower Anogenital Squamous Terminology Standardization Project for HPV-Associated Lesions: Background and Consensus Recommendations From the College of American Pathologists and the American Society for Colposcopy and Cervical Pathology. Arch Pathol Lab Med (2012) 136(10):1266–97. doi: 10.5858/arpa.LGT200570 22742517

[24] Sun H Shen K Cao D. Progress in Immunocytochemical Staining for Cervical Cancer Screening. Cancer Manag Res (2019) 11:1817–27. doi: 10.2147/CMAR.S195349 PMC6391129 30863187

[25] Li M Yang J Liu K Yang J Zhan X Wang L. P16 Promotes Proliferation in Cervical Carcinoma Cells Through CDK6-HuR-IL1A Axis. J Cancer (2020) 11(6):1457–67. doi: 10.7150/jca.35479 PMC6995400 32047552

[26] Scholzen T Gerdes J. The Ki-67 Protein: From the Known and the Unknown. J Cell Physiol (2000) 182(3):311–22. doi: 10.1002/(SICI)1097-4652(200003)182:3<311::AID-JCP1>3.0.CO;2-9 10653597

[27] Schmitz M Eichelkraut K Schmidt D Zeiser I Hilal Z Tettenborn Z. Performance of a DNA Methylation Marker Panel Using Liquid-Based Cervical Scrapes to Detect Cervical Cancer and Its Precancerous Stages. BMC Cancer (2018) 18(1):1197. doi: 10.1186/s12885-018-5125-8 30509219 PMC6276155

